# Intrauterine transfusion in 103 fetuses with severe anemia caused by parvovirus infection. A multicenter retrospective study

**DOI:** 10.1007/s00404-022-06712-z

**Published:** 2022-08-02

**Authors:** Philipp Kosian, Astrid Hellmund, Annegret Geipel, Rainer Bald, Otilia-Maria Geist, Paul Böckenhoff, Jorge Jimenez-Cruz, Maria Deja, Brigitte Strizek, Christoph Berg, Ulrich Gembruch

**Affiliations:** 1 Department of Obstetrics and Prenatal Medicine, University Hospital Bonn, Bonn, Germany; 2 Praxis Für Pränatalmedizin Bonn, Bonn, Germany; 3 Department of Gynecology and Obstetrics, Klinikum Leverkusen, Leverkusen, Germany; 4 Division of Prenatal Medicine and Gynecologic Sonography, Department of Obstetrics and Gynecology, University of Cologne, Cologne, Germany

**Keywords:** Fetal therapy, Intrauterine transfusion, Fetal infection by parvovirus B19, Fetal anemia, Fetal hydrops

## Abstract

**Purpose:**

Evaluating procedure-related complications and perinatal outcomes after intrauterine transfusion (IUT) before or after 20^+0^ weeks of gestation in fetuses with severe anemia due to intrauterine human parvovirus B19 infection.

**Methods:**

A retrospective study investigating fetuses requiring IUT for fetal Parvo B19 infection in two tertiary referral centers between December 2002 and December 2021. Procedure-related complications, intrauterine fetal death (IUFD), and perinatal outcome were correlated to gestational age (GA) at first IUT, the presence of hydrops and fetal blood sampling results.

**Results:**

A total of 186 IUTs were performed in 103 fetuses. The median GA at first IUT was 19^+3^ (13^+0^–31^+4^) weeks of gestation. IUFD occurred in 16/103 fetuses (15.5%). Overall survival was 84.5% (87/103). Hydrops (*p* = 0.001), lower mean hemoglobin at first IUT (*p* = 0.001) and low platelets (*p* = 0.002) were strongly associated with IUFD. There was no difference observed in fetuses transfused before or after 20^+0^ weeks of gestation.

**Conclusion:**

IUT is a successful treatment option in fetuses affected by severe anemia due to parvovirus B19 infection in specialized centers. In experienced hands, IUT before 20 weeks is not related to worse perinatal outcome.

## What does this study add to the clinical work

**Table:** 

Anemia due to parvovirus B19 infection can be successfully treated in experienced centers from the early second trimester with similar complications rates < 20^+0^ and ≥ 20^+0^ weeks of gestation. Overall, survival to delivery is high but factors associated with non-survival are fetal hydrops, the severity of anemia and thrombocytopenia.	

## Introduction

Parvovirus B19, a single-stranded round DNA virus is responsible for erythema infectiosum, a common illness observed in children. In most cases the infection will be asymptomatic, sometimes patients experience flu-like symptoms, the above-mentioned erythema infectiosum or arthropathies. In cases of intrauterine infections, parvovirus B19 attacks hematopoietic system cells, endothelial cells, placental cells, fetal liver and heart cells by binding to the P antigen. Erythroid progenitors in the bone marrow are targeted by the virus, inducing a shorter half-life of fetal red blood cells. This mainly affects hematopoiesis during the hepatic stage, consecutively leading to hemolysis and red blood cell aplasia [1–5]. Due to suppression of platelet precursors and direct cytotoxic effects on megakaryocytes, fetal thrombocytopenia is also commonly seen in fetuses affected by parvovirus B19 infection [6, 7]. The transplacental transmission rate is between 17 and 33%. Fetal anemia and hydrops are usually observed between 3 and 12 weeks after maternal infection. Fetal loss rate is reported at around 9% [8, 9]. According to a large observational study, most intrauterine fetal deaths (IUFD) are observed up to 4 weeks after maternal infection. Fetal losses occurred only in those infected before 20 weeks. The highest risk of IUFD was observed in infections between 9 and 16 weeks, and the highest risk for hydrops in infections between 13 and 20 weeks [8]. A recent systematic review and meta-analysis exploring the outcome of fetuses affected by parvovirus B19 infection included 35 observational studies and found a spontaneous resolution of the infection in 58.2% of the cases in non-hydropic fetuses [10]. Spontaneous resolution, however, was rare in hydropic fetuses (5%). Hydrops was associated with an increased risk for intrauterine or perinatal death. In this systematic review, IUT was performed in 78.7% of hydropic and 29.6% of non-hydropic fetuses. Fetal loss after IUT was higher in hydropic fetuses [10]. Fetal anemia is diagnosed by Doppler ultrasound measurement of the peak systolic velocity in the middle cerebral artery (MCA-PSV). This measurement is possible from the late first and early second trimester, in combination with other sonographic signs such as nuchal/generalized skin edema, echogenic bowel, cardiomegaly, placentomegaly and polyhydramnios [11]. Most fetuses do not develop relevant anemia or hydrops within the first 12 weeks after seroconversion and therefore do not need intrauterine transfusion. However, for severely anemic fetuses an IUT represents an important and in most cases life-saving treatment [12].

Data on intrauterine treatment of parvovirus B19-related anemia are limited [10, 13–17]. Parvovirus B19 often affects fetuses in the early second trimester but published data is mainly from IUTs performed after 20 weeks of gestation. We have previously published our experience of IUTs before 20 weeks of gestation [17]. Overall survival was 80.0% (44/55). Hydrops was present in 38.2% (21/55) and was strongly associated with IUFD (*p* = 0.001). There was no difference with regard to IUFD, hydrops, or hemoglobin concentration at first IUT in fetuses with transfusion before or after 16^+0^ weeks [17]. The aim of this study was to compare outcomes in pregnancies with severe anemia caused by parvovirus B19 infection and treated by IUT before or after 20^+0^ weeks of gestation.

## Materials and methods

This retrospective study included in total 103 fetuses that underwent an IUT due to parvovirus B19 infection at any time during pregnancy in two tertiary centers between December 2002 and December 2021. Maternal characteristics including gestational age (GA) at first IUT, fetal sonographic manifestations, fetal blood sampling results, details of IUT procedures, procedure-related complications, and perinatal outcome data were analyzed.

Fetal anemia was assessed by pulsed wave Doppler ultrasound evaluation of the peak systolic velocity in the middle cerebral artery (MCA-PSV) [18, 19]. In all cases a detailed anatomic scan was performed and presence or absence of polyhydramnios, placentomegaly, tricuspid regurgitation, cardiomegaly, ascites, echogenic bowel and skin edema were evaluated. The diagnosis of fetal infection was confirmed either by the quantitative viral load in fetal blood by polymerase chain reaction (PCR) in 68/103 fetuses (66.0%) or by fetal sonographic suspicion of severe anemia in combination with maternal IgM antibodies. In cases with an elevated MCA-PSV above 1.5 multiples of the median (MoM), an IUT was planned [19]. For fetuses between 12^+0^ and 14^+6^ weeks of gestation reference values are described in the publication by Hellmund et al. [17].

Fetal blood sampling was possible in 169/186 IUTs and a complete blood count was performed. Severe thrombocytopenia was considered if < 50 platelets/nl. If fetal blood sampling was not successful (*n* = 17), the decision on the amount of blood transfused was solely based on the estimated fetal weight (without hydrops). A fetal weight adapted volume of packed red blood cells (PRBCs) (0 rhesus-negative, cross-matched, cytomegalovirus-negative, irradiated, mean hemoglobin concentration 26.1 g/dl) was administered during the same intervention.

All the procedures were conducted by experienced operators of the two centers. Spinal needles of 20–25 G were used. To not cause volume overload, 30–50 ml PRBCs/kg estimated fetal weight were transfused. Antenatal corticosteroids to reduce the incidence and severity of respiratory distress syndrome were not administered prior to IUTs. The necessity for additional IUTs depended on the hemoglobin concentration after IUT and echocardiographic and Doppler sonographic results.

The next day, an ultrasound examination with Doppler was performed. Depending on the results, further follow-up was planned. The total number of transfusions per fetus and the interval between IUTs were evaluated. The perinatal outcome of survivors and non-survivors were compared.

Bleeding from the puncture site, dislocation of the needle, unsuccessful puncture, fetal bradycardia or IUFD within 24 h after IUT were categorized as procedure-related complications.

To investigate the hypothesis that earlier transfusions cause more complications, the study group was divided into fetuses transfused < 20^+0^ weeks of gestation (57 fetuses) and fetuses treated at ≥ 20^+0^ weeks of gestation (46 fetuses). Survival rate was also compared in IUTs < 16^+0^ and ≥ 20^+0^ weeks of gestation.

Part of the IUTs < 20^+0^ weeks of gestation have been previously published [15].

Data analysis was performed with IBM SPSS software version 27 (SPSS Inc., Chicago, IL, USA). Differences between subgroups were calculated with the Fisher exact test, Mann- Whitney-*U*-Test or Pearson correlation coefficient wherever appropriate. A *p *value < 0.05 was considered significant.

Patient data, technical aspects of IUT, complications, fetal blood sampling results and perinatal outcomes were collected from the Viewpoint Database Version 5 and Orbis Version 08.04.37.21, the electronic hospital information system.

Ethical review and approval were waived in view of the retrospective nature of the study.

## Results

103 patients underwent intrauterine transfusion for fetal anemia due to parvo B19 infection during the study period. In all cases, an increased MCA-PSV above 1.5 MoM was detected. 44/103 (42.7%) of fetuses were hydropic. Additional sonographic findings were tricuspid regurgitation (*n* = 53; 51.5%), ascites (*n* = 51; 49.5%), cardiomegaly (*n* = 42; 40.8%), pericardial effusion (*n* = 38; 36.9%), echogenic bowel (*n* = 32; 31.1%), skin edema (*n* = 31; 30.1%), placentomegaly (*n* = 33; 32.0%) and polyhydramnios (*n* = 25; 24.3%) whereas pleural effusion (*n* = 11; 10.7%) was less common. Reasons for referral were maternal seroconversion regarding human parvovirus B19 (*n* = 41; 39.8%), ascites/pericardial effusion or presence of fetal hydrops (*n* = 37; 35.9%), suspected maternal parvovirus exposure or maternal fever (*n* = 11; 10.7%), suspected fetal anemia (*n* = 9; 8.7%) and fetal echogenic bowel (*n* = 5; 4.9%) (Table 1).

**Table 1:** Characteristics of cases with parvovirus B19 infection and intrauterine transfusion (IUT)

**Total number of fetusus**	103
Number of IUTs (total, *n* = 186)	
1	46 (44.7%)
2	39 (37.9%)
3	12 (11.6%)
4	5 (4.8%)
5	0
6	1 (1%)
**Route of transfusion**	
Intravascular	167 (89.8%)
Intraperitoneal	13 (7.0%)
Intracardiac	6 (3.2%)
Weeks of gestation at first IUT (median)	19^+3^ (13^+0^- 31^+4^)
IUTs < 20 + 0 weeks	100 (53.8%)
IUTs ≥ 20 + 0 weeks	86 (46.2%)
Mean interval between 1st and 2nd IUT, days	6.6
Mean interval between 2nd and 3rd IUT, days	13.1
Mean interval between 1st and last IUT, days	12.3
Hydrops	44 (42.7%)
**Placental site**	
Anterior wall	57 (55.3%)
Posterior wall	46 (44.7%)
Fetal Death	16/103 (15.5%)
Overall Survival	87/103 (84.5%)
Fetal Loss Rate at 1st IUT	12/103 (11.7%)
Fetal Loss Rate at follow-up IUTs	4/57 (7.0%)

A total of 186 IUTs were performed in 103 fetuses between 13^+0^ and 31^+4^ weeks of gestation. Up to six subsequent IUTs were required in 57/103 fetuses (55.3%). Median gestational age at first IUT was 19^+3^ weeks. 57 fetuses (55.3%) were transfused before and 46 (44.7%) after 20^+0^ weeks.

The most common access was intravascular (in the umbilical vein) in 89.8% (*n* = 167) and the most common site of transfusion was the placental cord insertion (81.7%); an intraperitoneal approach was necessary for 7% of IUTs. Intracardiac IUT was performed in six cases (3.2%) as a rescue procedure and in one case primarily due to difficult access. Peritoneal access was significantly more common in transfusions before 20^+0^ weeks of gestation (*p* = 0.006). Spinal needles of 20–25 G were used. In 174/186 IUTs this information was available. The 20G needle was used in 55 (31.6%), the 22G needle in 108 (62.1%) and the 25G needle in 11 (6.3%) IUTs.

**Fig. 1:**
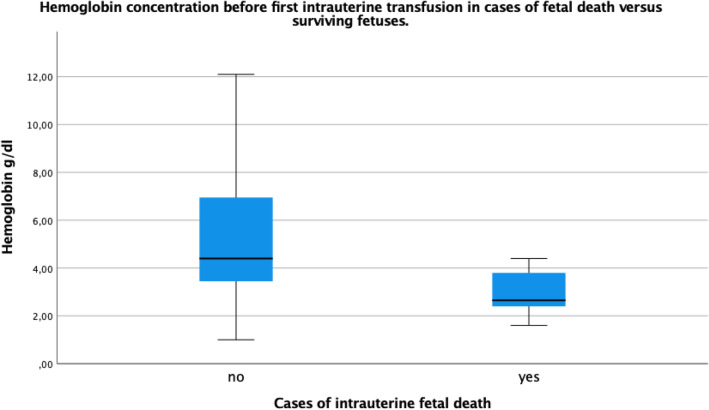
Hemoglobin concentration in g/dl before first intrauterine transfusion in cases of surviving versus non-surviving fetuses

The hemoglobin concentration at the first IUT was available in 90/103 fetuses (87.4%) and in 90.3% of all procedures (168/186), information on platelet count was available in 75 fetuses (72.8%). The mean Hb concentration before the first IUT was 5.0 g/dl (0.3–12.1 g/dl, SD 2.58). Mean hemoglobin concentration before the first IUT was significantly lower in non-survivors (2.63 g/dl, SD 1.16) than in survivors (5.3 g/dl, SD 2.55) (*p* = 0.001) (Fig. 1).

**Fig. 2:**
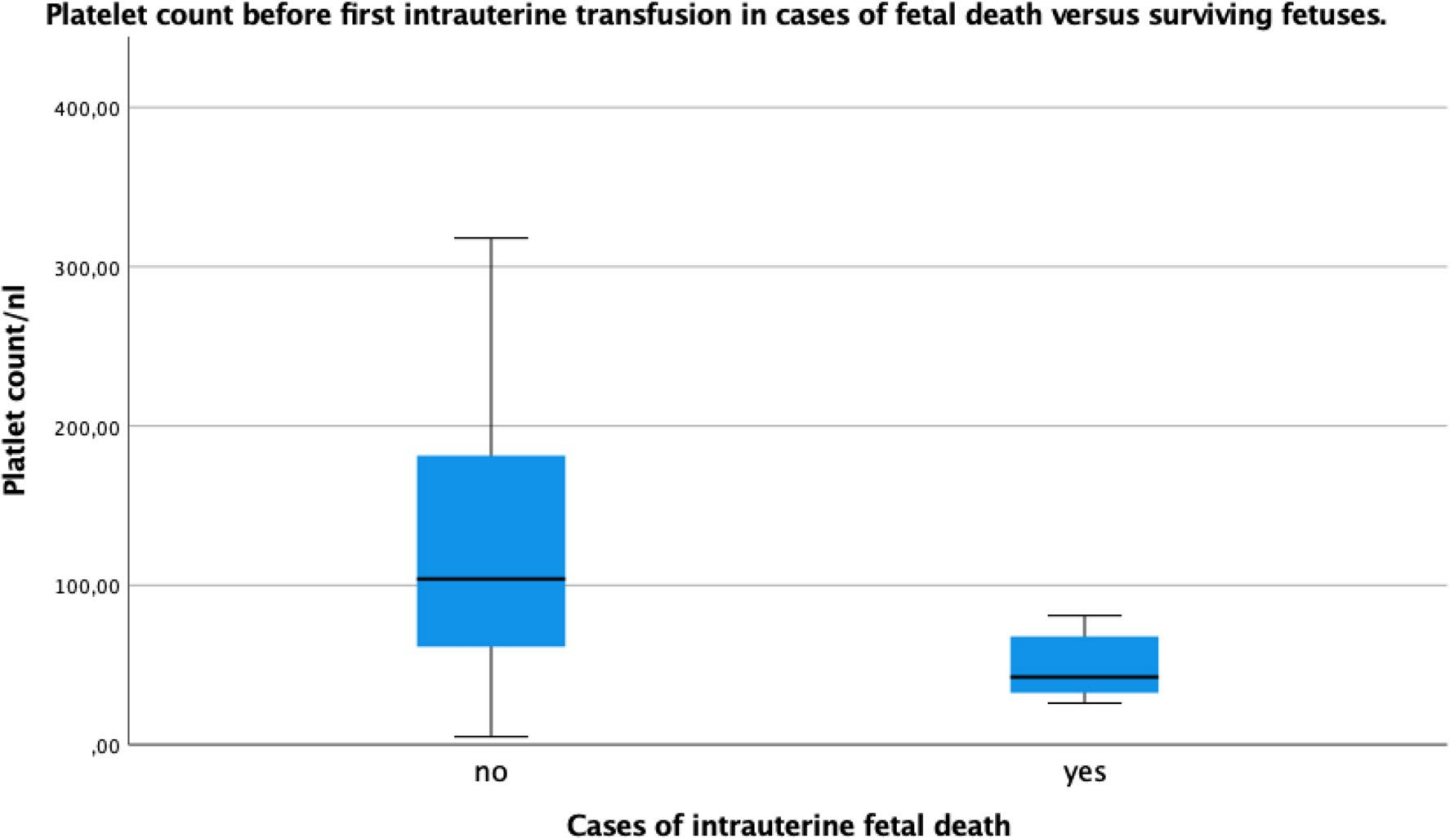
Platelet count before first intrauterine transfusion in cases of surviving versus non-surviving fetuses

Thrombocytopenia below 100 platelets/nl was present in 37/75 fetuses (49.3%). The platelet count was significantly lower in non-survivors (*p* = 0.02) (Fig. 2). Severe thrombocytopenia below 50 platelets/nl was much rarer in the survivor group (12/67;17.9%) than in the non-survivor group (5/8; 62.5%) (*p* = 0.013). Platelets were given in 10 of the 186 (5.4%) transfusions in case of thrombocytopenia according to the operators’ choice and were not administered routinely. We also observed a correlation between the hemoglobin and thrombocyte concentration before the first IUT (*p* = 0.001).

The overall survival rate was 84.5% (87/103). Sixteen (15.5%) IUFD occurred in 103 transfused fetuses. The IUFD rate after the first transfusion was 11.7% (12/103). In follow-up IUTs, fetal loss was rare (7.0%; 4/57). In 14 fetuses, death was noted during transfusion or within 24 h; in the other 2, death was detected on follow-up. Hydrops (*p* = 0.001) and low hemoglobin at first IUT (*p* = 0.001) were significantly associated with IUFD. Furthermore, posterior placental localization was associated with fetal loss (68.8%, 11/16 fetuses; *p* = 0.05). Prior to IUFD in these fetuses, dislocation of the needle (*n* = 2), bleeding from the puncture site (*n* = 3) and bradycardia during IUT (*n* = 1) were observed. Maternal body mass index on the other hand did not show any influence on procedure-related fetal loss (*p* = 0.97) (Table 2).

**Table 2:** Procedure-related complications < 20 + 0 and ≥ 20 + 0 weeks of gestation

Procedure-related complications	< 20 + 0 weeks	≥ 20 + 0 weeks
Overall (*n*, %)	15 (15%)	9 (10.5%)
Bradycardia (*n*)	1	1
Bleeding from puncture site (*n*)	2	1
Unsucessfull puncture (*n*)	1	1
Dislocation of needle (*n*)	2	1
Fetal death within 24 h (*n*)	9	5

Complications such as IUFD within 24 h, bradycardia, bleeding from the puncture site, contractions, dislocation of the needle or unsuccessful puncture occurred in 24/186 IUTs (12.9%). The median year of IUTs performed was 2009. To investigate if a learning curve exists, complication rates before and after 2009 were compared but did not differ significantly (*p* = 0.801). All operators were experienced and had performed at least 50 fetal blood samplings of the umbilical vein prior.

There was a lower survival rate in fetuses undergoing their first IUT < 20^+0^ weeks of gestation (80.7%) compared to ≥ 20^+0^ weeks of gestation (89.1%), but this did not reach statistical significance (*p* = 0.284). No differences in survival rate could be detected if IUTs < 16^+0^ (73.3% survival) and ≥ 20^+0^ (89.1% survival) weeks of gestation were compared (*p* = 0.204). Factors associated with IUFD after IUT are hydrops, mean hemoglobin before first IUT, mean platelet count, severe thrombocytopenia and posterior placental position (details Tables 3, 4).

**Table 3:** Characteristics of survivors and non-survivors in transfused fetuses

Characteristics	Survivor	Non-survivor	*P *value
Hydrops (%, *n*)	35.6% (31)	81.3% (13)	0.001
Median gestational age at 1st IUT	19^+2^	18^+4^	0.18
Mean hemoglobin before 1st IUT, g/dl	5.3 (1–12.1)	2.63 (0.3–4.4)	0.001
Mean platelet count/nl	126.6 (5–318)	49.1 (26–81)	0.002
Severe thrombocytopenia (< 50 platelets/nl) (%, *n*)	17.9% (12)	62.5% (5)	0.013
Posterior placental localization (%, *n*)	40.2% (35)	68.8% (11)	0.05
Mean maternal BMI	24.6 (17.6–37.1)	24.8 (19–33.2)	0.97

*p *value < 0.05 is considered significant

**Table 4:** Characteristics of fetuses with parvovirus B19 infection transfused < 20 + 0 and ≥ 20 + 0 weeks of gestation

Characteristics	< 20 + 0 weeks	≥ 20 + 0 weeks	*p *value
Hydrops (%, *n*)	38.6% (22)	47.8% (22)	0.42
Mean hemoglobin before 1st IUT, g/dl	5.0 (0.3–10.7)	4.94 (1.1–12.1)	0.66
Survivor (%, *n*)	80.7% (46)	89.1% (41)	0.28
Non-survivor (%, *n*)	19.3% (11)	10.9% (5)	0.28
Number of IUTs (%, *n*)			0.39
1	50.9% (29)	36.9% (17)	
2	29.8% (17)	47.8% (22)	
3	12.3% (7)	10.9% (5)	
4	7% (4)	2.2% (1)	
5	0	0	
6	0	2.2% (1)	

*p *value < 0.05 is considered significant

## Discussion

Our study investigated 103 fetuses affected by severe parvovirus B19 infection requiring transfusion. We found an overall survival rate of 84.5% (87/103 fetuses).

Survival rates in alloimmune fetal anemia vary between 77 and 97%, depending on GA at transfusion [20–22]. Survival rates for fetuses transfused at < 22 weeks due to alloimmunization are reported between 76 and 80% [23–25]. A large retrospective study analyzing 1678 IUTs in alloimmune fetal anemia observed fetal demise more often < 20^+0^ (17.0%, survival 83%) than ≥ 20^+0^ weeks of gestation (1.9%, survival 98.1%) [22]. The necessity for transfusion in fetal anemia due to parvovirus B19 infection usually occurs earlier in pregnancy compared to red blood cell alloimmunization. Additionally, at the time of diagnosis and IUT more fetuses are hydropic and severely anemic compared to fetuses affected by red blood cell alloimmunization. In a review, perinatal survival after IUT was 66.7–72.2% in parvovirus B19-infected fetuses versus 80.0–93.5% in cases of red blood cell alloimmunization [26]. Survival rates in cases of parvovirus infection have been reported between 33 and 100% in the literature [6, 8, 13–16, 27–39]. GA varied between studies, but cases before 20 weeks of gestation were less commonly included. Part of our data already published investigating IUTs performed before pregnancy week 20^+0^, found a survival of 80.0% (44/55) [17]. In our study, we also observed a trend towards lower survival < 20^+0^ weeks of gestation (80.7%) compared to ≥ 20^+0^ weeks of gestation (89.1%), but this did not reach statistical significance (*p* = 0.284). 

Intravascular transfusion is considered the standard treatment for severe fetal anemia, regardless of its different etiologies. In our study, an intravascular access was possible in the majority (89.8%) of all IUTs. There was a significantly higher rate of peritoneal transfusions before gestational week 20^+0^ as described in the literature [40]. In a small study investigating an intracardiac approach between 17 and 23 weeks of gestation in fetuses with hydrops due to red blood cell alloimmunization (3 cases) or parvovirus B19 infection (5 cases), a survival rate of 75% was reported [34]. In our case series, however, in 6/186 IUTs (3.2%) an intracardiac transfusion was necessary leading to IUFD in all cases. 5/6 cases were as a rescue procedure in hydropic fetuses.

Thrombocytopenia is related to fetal hemoglobin count in anemic and hydropic fetuses as described by Segata et al. [6]. They described 11 hydropic fetuses of whom 75% were thrombocytopenic [6]. In our series, we observed thrombocytopenia in 68% of fetuses and 22.7% had severe thrombocytopenia. The platelet count was significantly lower in fetuses with hydrops compared to non-hydropic fetuses (*p* = 0.0001) and thrombocytopenia was also more commonly seen in non-survivors (*p* = 0.002). There was no difference observed between fetuses transfused in < 20^+0^ and ≥ 20^+0^ weeks of gestation (*p* = 0.126). We also observed a correlation between the hemoglobin and thrombocyte concentration before the first IUT as described in the literature [35] (Table 5).

**Table 5:** Literature review of transfused fetuses with parvovirus B 19 infection [6, 8, 13–16, 27–37, 39]

Study (first author)	Transfused case	GA at first transfusion (weeks of gestation)		Hydrops	Cases with severe thrombocytopenia	Survival rate (%; n)
		< 20 + 0	_≥ 20 + 0_			
Smoleniec, 1994 [15]	5	1	4	5	1	60 (3/5)
Fairley, 1995 [27]	12	n.a	n.a	7	n.a	75 (9/12)
Cameron, 1997 [28]	3	0	3	3	n.a	33 (1/3)
Odibo, 1998 [29]	3	1	2	3	n.a	100 (3/3)
Rodis, 1998 [30]^a^	164	n.a. (18–32)	n.a	137	n.a	83.5 (137/164)
Forestier, 1999 [31]	7	1	6	7	2	85.7 (6/7)
Schild, 1999 [14]	30	3	27	30	11	80 (24/30)
Enders, 2004 [8]	16	n.a	n.a	16	n.a	87.5 (14/16)
Segata, 2007 [6]	11	3	8	8	7	72.7 (8/11)
De Haan, 2008 [37]	30	2	28	30	14	77 (23/30)
Simms, 2009 [13]	8	n.a. (18–27)	n.a	8	2	62.5 (5/8)
Chauvet, 2011 [32]	19	n.a	n.a	19	n.a	47 (11/19)
Garabedian, 2014 [33]	13	0	13	5	n.a	76.9 (10/13)
Macé, 2014 [16]	17	0	17	17	9	76 (13/17)
Melamed, 2015 [35]	29	4	25	25	11	75.9 (22/29)
Mackie, 2015 [34]	5	2	3	5	2	60 (3/5)
Zavattoni, 2016 [36]	9	0	9	9	n.a	66.7 (6/9)
Sanchéz-Durán, 2020 [39]	10	n.a	n.a	8	n.a	80 (8/10)
Current Study^b^	103	57	46	44	17	84.5 (87/103)
Total	494	74/265^c^	191/265^c^	386/494	76	79.5 (393/494)

*GA* gestational age, *n.a.* not available

^a^Multicenter survey

^b^55 cases were previously published [17]

^c^The timing of the first IUT could be classified as < 20 and ≥ 20 weeks of gestation in 265 of the 494 cases

Data on neurodevelopmental outcome after fetal parvovirus B19 infection are heterogeneous. Dembinski et al. reported normal neurodevelopment after hydrops and IUT in 20 survivors with follow-up ranging from 13 months to 9 years of age [41] whereas Nagel et al. observed a delayed psychomotor development in 5 of 16 (32%) surviving infants at 1 to 42 months of age. At the median age of five years (range 1.5–13 years), De Jong et al. from the same center reported neurodevelopmental impairment in only 11% (2/28) of hydropic cases treated with IUT [42]. In our study, no follow-up data on postnatal neurodevelopment or cardiac function was available. Another limitation of our study was its retrospective design and the lack of blood sample values in some cases of IUT, especially in those transfused before 20 weeks of gestation with a 25-G needle. Proof of fetal parvovirus B19 infection was not possible in these cases but was very probable due to maternal seroconversion, sonographic signs—all these fetuses were hydropic—and severely elevated MCA-PSV. IUTs were performed by very experienced operators/centers and therefore the high rate of survival especially in fetuses transfused before 20 weeks of gestation of pregnancy might not be transferrable to centers with less expertise in this field.

In summary, anemia due to parvovirus B19 infection can be successfully treated in experienced centers from the early second trimester with similar complication rates < 20^+0^ and ≥ 20^+0^ weeks of gestation. In the vast majority of fetuses (> 80%), one or two IUTs are sufficient to bridge the time until intrauterine recovery. Overall, survival to delivery is high. Factors associated with non-survival are fetal hydrops, the severity of anemia and thrombocytopenia. An intravascular approach is possible in most cases.

## Data Availability

Not applicable.
